# On the Perspirable Fluids

**Published:** 1800-08

**Authors:** 


### *On the Perspirable Fluids*; *by* Dr. Mitchill.

[Concluded from p. 9—13 of our last.]

" Thus a solution of an alkaline salt, or of a soap, detaches all *acids* from the cuticle and from clothes, as likewise from all oily or other connections they may have formed. And the same lixivial and saponaceous mixtures remove all *greasy* substances, by virtue of the stronger attraction they exert toward these, than these possess for the cuticle, or the garment worn next to it. On the cleansing of clothes, there is one fact worthy of being mentioned. Sailors, on West India voyages, when destitute of soap, frequently wash their clothes in water, WITH THE AID OF MOLASSES, as a substitute for soap. They find this practice answer pretty well. Sugar is known to decompound nitrous acid very readily. Is not the purification of mariners' clothes, in this manner, a circumstance of weight, to evince the decomposition of the septic acid they contain, by the saccharine ingredient of the molasses?

" Such is the detail of the principal constituent parts of that nastiness which accumulates on the skins and in the garments of human beings; and such the theory of the operation of alkalies &c. in making them clean. From a comparison of the facts relative to *perspiration* with those which respect *urine*, it will readily appear, that the two fluids very much resemble each other, as might naturally be supposed from the great connection there has been long known to exist between the two discharges. What is, therefore, true of the one, is, to most purposes, applicable to the other. In particular, the sweat as well as the urine contains *septon*, which often combines with oxygen, and forms that noxious compound, *septic acid*.

" Yet it must be remembered, that as extractive matter and acids are capable of solution in mere water, and require not the aid of alkalies to detach them, that washing the cuticle in cool, or, more readily, in warm water, removes every thing but the grease, and even loosens or liquefies a part of that. Water cleanses the cuticle or a shirt by exerting a greater attraction for the foul material sticking to them, than the skin or a garment does. For this purpose the water must be clean, or, at least, less tainted than the subject intended to be purified. Upon the same principle that *water* or *alkaline ley* cleanses the cuticle, a *clean shirt* will do the same; that is, by attracting or absorbing from it something foul or foreign. *Clean shirts*, *drawers*, and *stockings*, are, therefore, substitutes for *clean water:* and he who changes his linen very frequently, deterges the surface of the body *almost* as much as by bathing; and, perhaps, haps, for the purpofes of health, *quite* as much. This appears to be more certainly the cafe, becaufe, after wafhing in foap-fuds, a portion of alkali adheres to the cloth, unlefs it be well rinfed, and is inftantly ready to exert its neutralizing effect. A dry fhirt, thus wafhed, may be conceived as befprinkled with innumerable minute cryftals of potafh and foda.

"Pathologifts have obferved that *acid fweats* have fometimes occurred. In thefe cafes an acefcent difpofition, or acid acrimony prefent in the blood, at the critical period of fevers, and at other times, may tincture the fluids fecreted in the veffels of the fkin with fome of their peculiar qualities. This has been alleged to have taken place to fo high a degree as to turn *blue* litmus-paper to a *red*. It is not material to my argument whether this happens frequently or never. It is enough for my purpofe that the liquids excreted on the cuticle have a tendency to become acid, *after* being depofited there. If *acid fecretions* ever happen, they exhibit a ftronger cafe, and illuftrate the doctrine with additional force.

"II. Another general property of thefe vitiated or corrupted animal excretions is to rot the cloathing to which they adhere, and caufe it to wear out the fafter A fhirt, indeed, worn until its threads are fully impregnated with foulnefs, requires fo much foaking and friction, while in the wafh, that there can be no wonder if, in the operations of being made clean, it is almoft wrung or rubbed to pieces. But, independent of all this, it is well known that linen, and cloathing of all kinds, become very remarkably weaker by lying and remaining long in their dirt, without being ever handled by the laundrefs. There may be fuch a collection and concentration of animal matter, as not only to rot the cloth, but to convert it to an infectious fomes, and make it diffufe peftilential vapours around. Garments, too long worn, without being cleanfed from time to time, often grow *rotten* and *infectious* as they grow *nafty*. Alkalies remove the naftinefs, and, if applied difcreetly, in feafon, prevent the rottennefs, and check the infection. Every day's experience demonftrates their efficacy in thefe proceffes. The following fact may ferve as an exemplification of a very common occurrence: A gentleman, in the time of war, was taken captive, and compelled, by the enemy, to march on foot a great diftance, at a violent rate, during very hot weather. He fweated moft profufely. At the end of the march he was confined in prifon, and treated with great rigour. Being deprived of changes of raiment, and of the means of keeping himfelf clean, his fhirt, in a fhort time after his confinement, became rotten, and he fell fick of a fever. In this inftance, no doubt, both the cuticle and fhirt became impregnated ted with *septic poison*, manufactured from the *sweat*, from which both the *constitution* and the *clothing* were the sufferers. In this way the American and British sailors, who visit the West-Indies, *catch yellow fevers*, as they are called, without once going on shore, or having any communication with infected persons. Numberless facts prove that, even in the harbours, these fatal distempers are not *introduced from on shore*, *but bred and supported on board the vessels themselves*, in which the people fall sick, whence they are carried on shore to be cured.

"III. A third general effect of these septic productions, as was observed in the preceding paragraph, is *to injure the constitution.* This they do in two ways: 1. By a direct stimulant operation upon the part of the *true skin* to which they are applied, causing different forms of tinea, herpes, psora, elephantiasis, petechial and miliary eruptions, &c. &c. and, 2. By a direct stimulant operation upon the heart and sanguiferous vessels, after they are conveyed thither through the absorbents arising from the cuticular surface, causing various grades of febrile disturbance, of the intermittent, remittent, and continued forms, and inducing distempered motions in the lymphatic system, as they pass through its branches. All examiners of this subject should, however, recollect, that septic fluids, though they exist on the cuticle and in the blood-vessels, do not always stir up diseases; for the constitution may be, and frequently is, in such a state as to resist their tendency to produce morbid action; the reason of which seems to be, that the stimulus no longer irritates a constitution which is seasoned or habituated to it.

"IV. A fourth general property of these fluids, engendered in corruption, is to be volatilized by the heat of summer and autumn in the United States. The noxious quality of the vapours proceeding from putrefying collections of common animal matter is too well known to require mentioning in this place. When that other animal matter which constitutes perspiration and sweat, and accumulates in the cuticle and clothing to large amount, has undergone the changes to which it is prone, it is much disposed to assume the aërial form. Some portions of these exhaling vapours are often pungent, active, and of a deleterious quality. They are produced, generally speaking, not in the circulating blood, nor during the process of secretion, but *after their* deposition upon the cuticle and clothes, and aided by HEAT and MOISTURE, through a process similar to that which converts urine, and several other excrementitious substances, into offensive and noxious airs.

"Clothing, or merchandize of any kind, wherein noxious airs or fluids of the septic kind are generated or accumulated, is called a FOMES. In this way bills of paper-money paffing frequently from man to man, and becoming tainted with every thing that human hands can impart to them, have, in the city of New-York, degenerated to a *fomes*. So the fhoes and ftockings of that unclean perfon who fuffers feptic acid fo to accumulate about the toes and feet as to eat away the cuticle to the quick, may become a *fomes*. In like manner a feaman's cheft of dirty clothes, tainted with all kinds of animal exudations, may, during a voyage from Cape-Françoife to New-York, turn to a *fomes*, though not a particle of infection was borrowed from the atmofphere of St. Domingo, or from any perfon living or dying in it. By a procefs in no wife different, do feather beds, and beds of all kinds, that have been long laid upon, and thereby become thoroughly impregnated with perfpirable and other matter, acquire their unwholefome qualities, and turn to a *fomes*. Juft thus are clothes charged with perfpirable and other excrementitious matter, difcharged from the bodies of prifoners in jail, and of other perfons living in foul and confined places, converted to venom and peftilence, or to an infectious or contagious *fomes*. And it is not impoffible that a bale of cotton, or any fimilar material, may, by impregnation with animal matter, become a repofitory of mifchief, and a *fomes*.

"In the preceding paragraph I have introduced the word "fomes" as often as I conveniently could; and have done fo that I might fhew the impropriety of the term, and offer my objections to it. The *literal* meaning of *fomes*, in Latin, refers to that quality, in bodies, which makes them catch and retain fire very readily; fuch as punk, tinder, lint-ftock, and touch wood (rapuitque in *fomite* flammam—Virg. Æneid i. v. 180; in the note to which, in the Dauphin's edition, may be feen a blunder of the critics.) ACHATES ufed a *fomes* when he ftruck fire with flint, &c. *Metaphorically*, it denotes an incentive or motive to any action. While the belief prevailed, that infection was carried from place to place, like a fire-brand, and paffed from perfon to perfon, like a fpark, the word "fomes" was borrowed to exprefs, by a ftrong and illuftrative figure, the analogy between the fuppofed effects of fire and thofe of peftilence. As each of the fubftances juft named was a fomes of fire, fo a cheft of clothes, a blanket, a bed, or a bale of merchandife, was a fomes of peftilence. This mode of expreffion was thought to be highly philofophical and correct; infomuch, that when any one affirmed that an atom of contagious matter, received into a blanket, multiplied itfelf as a fpark of fire enlarges in a tinder-box, he was thought to exprefs himfelf fenfibly.

"But

" But it will appear, to any one who comprehends the preceding obſervations, that the ſimile, if it be intended for a ſimile, is imperfect; or the metaphor, if it is meant for a metaphor, is deceitful: for it has been ſhewn, that the thing called a *fomes* produces peſtilential matter without having a particle of infection (like a ſpark of fire, or even a bit of leaven) introduced into it from without; and it follows, of courſe, that all figurative expreſſions, calculated to uphold the idea *of foreign introduction* of common peſtilential matter, and its *derivation ab extrà*, are highly conducive to the continuance and propagation of error. It is high time this form of ſpeech was diſcontinued. This figurative expreſſion itſelf, and the unhappy aſſociation of ideas which ſprings from it, are the cauſe of a large proportion of the wrong-headedneſs now prevalent, concerning the diſtempers called infectious.

" Was not the malignant fever, or plague, which deſtroyed a great proportion of one thouſand nine hundred and forty-ſix convicts, on board the hulks in the Thames, (Colquhoun's Police of the Metropolis, chap. xii. p. 307) generated in the veſſels? And were there not local cauſes enough to excite it, without the importation and introduction of a fomes? Did not the peſtilential diſtemper which afflicted the Spaniſh captives at Wincheſter ariſe within the walls of their priſon, independent of any fomes introduced from without? Was not Hoſier's ſquadron, during the unfortunate expedition againſt Porto-Bello, diſmally diſabled by a diſtemper, bred on board and within the ſhips, wholly unaided by any thing like a fomes? And, if it was not ſuperfluous, I would aſk, whether malignant diſtempers did not harraſs, with their viſitations, almoſt all the great cities of Europe, and, indeed, of the world, until, by enforcing ſufficient laws againſt nuiſances, their inhabitants removed the cauſes?

" In all theſe inſtances, dreadful as ſome of them were, the cauſes of the diſtempers were engendered or manufactured on the ſpot. Theſe exciting cauſes were, in all, exactly ſimilar to that which dirties a ſhirt, or the commoneſt article of clothing. If there is any difference of moment, it is nearly this: in one caſe peſtilence is manufactured upon a *great* ſcale, and, in the other, upon a *ſmall* one. The ſame principle pervades both caſes. The laws of Nature operate uniformly in both: *and the ſame means that will cleanſe a ſhirt, will purify a ſhip or a jail.* Subſtances that ſubdue acids, ſuch as OILS, CALCAREOUS EARTH, AND SOLUTIONS OF ALKALINE SALTS, CAN ACCOMPLISH THE WORK.

" And if I interpret right that accurate mineralogiſt FERBER, (Travels through Italy, letter xi. p. 129, edit. by Raſpe) the the catacombs, near the cities of Naples and Rome, are cut through and into maſſes of *tufo-ſtone.* This ſeems to conſiſt chiefly of volcanic aſhes, hardened into a ſubſtance of ſufficient compactneſs, in many caſes, for building: and thoſe afforded by Veſuvius, whether grey, white, brown, or yellowiſh, contain *alkaline* or *calcareous* particles enough to effervesce with acids. Hence the preſervation of human bodies in thoſe ſubterranean chambers can be eaſily accounted for. The lime preſerved theſe as the ſoda preſerved the mummies. Spallanzani has accounted for the preſence of this alkaline matter. In his very intereſting travels, (Viaggi alle due Sicilie, &c. Pavia, 1792) he affirms, that a large tract of the Appenines, and the ſubſtratum of the volcanic country about Naples, conſiſt of the *carbonate of lime.* (tom. i. p. 109—114) And as he has ſhewn that ſundry volcanic productions, particularly the vitrified lavas of the Lipari Iſlands, contain *muriatic acid,* (tom. iii. p. 351) which, he thinks, is derived from the decompoſition of *common ſalt,* (p. 361) *veramente del mare iſteſſo penetrante per di ſotto i monti volcanici,* penetrating from the ſea itſelf, beneath volcanic mountains, does it not follow, that its *ſoda* muſt be combined wiih other foſſils, and, perhaps, with the tufo?

"There has been a number of tolerable conjectures on the preciſe nature of peſtilential matter. Beſides thoſe I have mentioned in other eſſays, I refer you now to the *Loimologia* of Hodges, a phyſician who reſided in the city of London during all the time of the plague of 1665. Obſerve how well he has gueſſed. In his § 1, on the Cauſe of a Peſtilence and a Contagion, he conſiders it as "ariſing chiefly from a change or corruption of the *nitrous ſpirit* in the air." (p. 32 and 37) The *nitrous ſpirit* of this writer, as well as of his countryman and predeceſſor Mayow, was evidently *vital* or *oxygenous air*; and, by its change or corruption, may be obviouſly underſtood, a combination of it with ſepton, the principal of putrefaction, or azote, the element hoſtile to life. To this change in the nitrous principle, Hodges aſcribes "blaſts upon trees and diſeaſes among cattle," as well as a peſtilence among mankind, (p. 41); and to ſomewhat of *an acid,* ſometimes produced in this way, he attributes the quality of rain-water to ſtain the clothing and ſkins of travellers in the equatorial regions of Africa, and "burn upon them, as it were peſtilential characters." (p. 42) He affirms, too, what I have often witneſſed, that there is a great ſimilitude between a peſtilence and a ſcorbutic habit, (p. 46); and declares, (p 49) that the convulſions and vellications of the ſtomach and alimentary canal are not owing to a putrefaction of of bile, nor an eruption of fire, as the manner of thinking then was, but to this "vitiated saline spirit." This author makes favourable mention (p. 171) of spirit of hartshorn, (ammoniac) given in doses of two scruples and one drachm.

"I shall, however, rest the argument here for the present. If the reasoning advanced establishes the principle, then the principle so established may be employed to explain innumerable other facts in Nature belonging to this head. Your sagacity will judge both of the rule and its application.

"Be assured I am yours,

"With high consideration and respect,

"SAMUEL L. MITCHILL."

